# Nanoconfined Crosslinked Poly(ionic liquid)s with Unprecedented Selective Swelling Properties Obtained by Alkylation in Nanophase-Separated Poly(1-vinylimidazole)-*l*-poly(tetrahydrofuran) Conetworks

**DOI:** 10.3390/polym12102292

**Published:** 2020-10-07

**Authors:** Tímea Stumphauser, György Kasza, Attila Domján, András Wacha, Zoltán Varga, Yi Thomann, Ralf Thomann, Balázs Pásztói, Tobias M. Trötschler, Benjamin Kerscher, Rolf Mülhaupt, Béla Iván

**Affiliations:** 1 Polymer Chemistry Research Group, Institute of Materials and Environment Chemistry, Research Centre for Natural Sciences, Magyar tudósok körútja 2, H-1117 Budapest, Hungary; kasza.gyorgy@ttk.hu (G.K.); pasztoi.balazs@ttk.hu (B.P.); 2 George Hevesy PhD School of Chemistry, Institute of Chemistry, Faculty of Science, Eötvös Loránd University, Pázmány Péter sétány 2, H-1117 Budapest, Hungary; 3 NMR Research Laboratory, Instrumentation Center, Research Centre for Natural Sciences, Magyar TudóSok Körútja 2, H-1117 Budapest, Hungary; domjan.attila@ttk.hu; 4 Biological Nanochemistry Research Group, Institute of Materials and Environment Chemistry, Research Centre for Natural Sciences, Magyar Tudósok Körútja 2, H-1117 Budapest, Hungary; wacha.andras@ttk.hu (A.W.); varga.zoltan@ttk.hu (Z.V.); 5 Freiburg Materials Research Center, University of Freiburg, Stefan-Meier-Str. 21, D-79104 Freiburg, Germany; yi.thomann@fmf.uni-freiburg.de (Y.T.); ralf.thomann@fmf.uni-freiburg.de (R.T.); tobias.troetschler@fmf.uni-freiburg.de (T.M.T.); benjamin.kerscher@fmf.uni-freiburg.de (B.K.); 6 Freiburg Center for Interactive Materials and Bioinspired Technologies (FIT), University of Freiburg, Georges-Köhler-Allee 105, D-79110 Freiburg, Germany; 7 Institute for Macromolecular Chemistry, University of Freiburg, Stefan-Meier-Str. 31, D-79104 Freiburg, Germany

**Keywords:** nanoconfined poly(ionic liquid), amphiphilic conetworks, poly(1-vinylimidazole), poly(tetrahydrofuran), nanophase-separated structure, selective swelling, superabsorbent

## Abstract

Despite the great interest in nanoconfined materials nowadays, nanocompartmentalized poly(ionic liquid)s (PILs) have been rarely investigated so far. Herein, we report on the successful alkylation of poly(1-vinylimidazole) with methyl iodide in bicontinuous nanophasic poly(1-vinylimidazole)-*l*-poly(tetrahydrofuran) (PVIm-*l*-PTHF) amphiphilic conetworks (APCNs) to obtain nanoconfined methylated PVImMe-*l*-PTHF poly(ionic liquid) conetworks (PIL-CNs). A high extent of alkylation (~95%) was achieved via a simple alkylation process with MeI at room temperature. This does not destroy the bicontinuous nanophasic morphology as proved by SAXS and AFM, and PIL-CNs with 15–20 nm d-spacing and poly(3-methyl-1-vinylimidazolium iodide) PIL nanophases with average domain sizes of 8.2–8.4 nm are formed. Unexpectedly, while the swelling capacity of the PIL-CN dramatically increases in aprotic polar solvents, such as DMF, NMP, and DMSO, reaching higher than 1000% superabsorbent swelling degrees, the equilibrium swelling degrees decrease in even highly polar protic (hydrophilic) solvents, like water and methanol. An unprecedented Gaussian-type relationship was found between the ratios of the swelling degrees versus the polarity index, indicating increased swelling for the nanoconfined PVImMe-*l*-PTHF PIL-CNs in solvents with a polarity index between ~6 and 9.5. In addition to the nanoconfined structural features, the unique selective superabsorbent swelling behavior of the PIL-CNs can also be utilized in various application fields.

## 1. Introduction

Nanoconfined material structures, including polymers as well, due to the wide range of unique structural features, properties, and effects arising under nanocompartmentalized conditions, have created significant worldwide interest over the last couple of years (see e.g., references [1,2,3,4,5,6] and references therein). The recently emerged polymer conetworks, especially amphiphilic conetworks (APCNs), composed of covalently or supramolecularly bonded, otherwise immiscible, hydrophilic and hydrophobic polymer chains, belong to such nanophase-separated materials with mutually nanoconfined macromolecular components [7,8,9,10,11,12,13,14,15,16,17,18,19,20,21,22,23,24,25,26,27,28,29,30,31,32,33,34,35,36,37,38,39,40,41]. Considering that polymers containing imidazole rings, which also play a pivotal role in the major biopolymers and compounds of living organisms, such as DNA, RNA, proteins, enzymes, hormones, vitamins, etc., provide various advantageous properties, like water solubility, strong metal ion coordinating ability, protonation, and alkylation possibilities, we have recently explored the synthesis, nanophasic structure, and properties of poly(1-vinylimidazole)-*l*-poly(tetrahydrofuran) (PVIm-*l*-PTHF) and poly(1-vinylimidazole)-*l*-poly(propylene glycol) (PVIm-*l*-PPG) conetworks (-*l*- stands for “linked by”) [8,34,35,36,37,38,39,40]. In addition to revealing the basic features of the synthesis and swelling behavior of these conetworks, it was found by us that the PVIm-*l*-PTHF conetworks possess nanophase-separated morphology with ~7–20 nm average domain sizes and bicontinuous (cocontinuous), i.e., a mutually nanoconfined domain structure in an unprecedented broad range of composition (~25–60% weight fraction of PVIm) [39]. This unique cocontinuous nanoconfined phase structure and the imidazole rings in the PVIm-*l*-PTHF conetworks provide various possibilities to create several novel materials which have not existed before.

Another class of imidazole-based polymers belongs to poly(ionic liquid)s (PILs), that is macromolecules with ionic liquid (IL) moieties. Due to the challenging tasks of the synthesis, revealing the structure–property relationships, and the broad range of special application possibilities, PILs have been intensively investigated in recent years (see e.g., references [41,42,43,44,45,46,47,48,49,50,51,52,53,54,55,56,57,58,59,60,61,62,63,64,65,66,67,68,69,70,71,72,73,74,75,76,77,78,79] and references therein). PILs may find applications in energy production as proton [57], electron [58,59], or Li-ion [60,73] conducting materials; as metal free [61] and metal containing [62] catalysts; gas separation membranes [63,64]; DNA isolation microspheres [65] etc. PILs are prepared either by polymerization of ionic liquid-type monomers [42,43,44,45,46,47,48,49,50,51,52,53], or less frequently by polymer analogous postmodification of suitable polymers [66,67,68] as shown, for instance, recently by the synthesis of PVIm-based PIL brushes via termination of oxazoline polymerization by PVIm homopolymer [68]. Although several applications require crosslinked PILs, PIL networks have not gained broad interest yet. The crosslinking usually takes place by copolymerizing IL monomers with conventional crosslinking agents, such as divinylbenzene and ethylene glycol dimethacrylate [43,69,70,71] or by bis-vinylimidazolium compounds [72,73]. In a few cases, low molecular weight poly(ethylene glycol) (PEG) di(meth)acrylate oligomers were used as crosslinkers in the preparation of PIL networks [41,74,75,76,77,78,79]. Although these crosslinked PILs can be considered as PIL conetworks with the PEG oligomer crosslinkers, the conetwork aspects have not been investigated so far, most likely because of the relatively low PEG contents in these materials.

While nanoconfined low molecular weight ionic liquids have been widely investigated (see e.g., reference [80] and references therein), systematic research on nanoconfined poly(ionic liquid) chains or chain segments—with the exception of the polymerization of vinylimidazolium ionic liquid monomers in aluminum oxide nanochannels with 18–150 nm pore diameter by Kaminski and coworkers [81,82], and the effect of confinement on the ionic conductivity of PIL block copolymers by Segalman et al. [83]—have not been reported so far according to the best of our knowledge. Taking into account the presence of poly(1-vinylimidazole) in the nanophase-separated PVIm-*l*-PTHF conetworks as one of its components, this inspired us to explore the possibility of the synthesis of nanoconfined PIL conetworks by alkylation of the imidazole pendant groups of the PVIm nanodomains in the conetworks. These challenging studies have raised several aspects of such a process unexplored so far, such as whether alkylation can be carried out in the nanoconfined PVIm phase, what would be the extent of alkylation in the nanophasic conetworks, and what would be the effect of this process on the fundamental structural characteristics, especially on keeping or destroying the nanophase-separated structure by alkylation of the PVIm phase, and how the swelling capabilities of the resulting PIL conetworks (PIL-CNs) would change in comparison to the starting nanophasic PVIm-*l*-PTHF amphiphilic conetworks. Herein, we report on the synthesis, structural investigations, and the unexpected swelling behavior of PIL conetworks obtained by converting the imidazole rings to imidazolium ionic liquid moieties inside the nanostructured PVIm-*l*-PTHF conetworks.

## 2. Materials and Methods

### 2.1. Materials

1-Vinylimidazole (VIm) (Sigma-Aldrich, St. Louis, MO, USA) was vacuum distilled prior to use. Hydroxyl-telechelic poly(tetrahydrofuran) (PTHF, Teratane 2000, M_n_ = 2000 g/mol, Sigma-Aldrich, St. Louis, MO, USA) was purified by precipitation from dichloromethane into hexane, then dried in a vacuum at 35 °C. 2,2′-Azobis-isobutyronitrile (AIBN; Sigma-Aldrich, St. Louis, MO, USA) was recrystallized from methanol before use. Methacryloyl chloride (MACl, 97%, Honeywell Fluka, Charlotte, NC, USA) was freshly distilled prior to use. Dichloromethane (DCM, 99%, Molar Chemicals, Halásztelek, Hungary) and ethanol (EtOH, 99.8%, VWR International, West Chester, PA, USA) were purified by distillation and kept under nitrogen. Triethyl amine (TEA), neutral aluminum oxide (Al_2_O_3_) (both from Sigma-Aldrich, St. Louis, MO, USA), and magnesium sulfate (MgSO_4_, Molar) were used as received. Tetrahydrofuran (THF, 99.5%), methanol (MeOH, 98.5%), N,N-dimethylformamide (DMF, 99.8%), dimethylsulfoxide (DMSO, 99%), acetonitrile (99%) (all from VWR International, West Chester, PA, USA), N-methyl-pyrrolidone (NMP, 99.5%) and bioreagent water (Sigma Aldrich, St. Louis, MO, USA), ethyl acetate (99%), and hexane (Hex, 51%) (purchased from Molar Chemicals, Halásztelek, Hungary) were used as received. Silver behenate was purchased from Alfa Aesar (Thermo Fischer Scientific, Ward Hill, MA, USA) and used without further purification.

### 2.2. Synthesis of Methacrylate-Telechelic Poly(tetrahydrofuran)

Methacrylate-telechelic poly(tetrahydrofuran) (MA-PTHF-MA) was synthesized by a reaction between hydroxyl-telechelic PTHF and methacryloyl chloride, as described earlier [34]. Briefly, 6.98 g hydroxyl-telechelic PTHF was dissolved in 150 mL of dichloromethane, and then, the oxygen was removed with a nitrogen purge for one hour. After cooling with isopropanol/dry ice, first 27.8 mL triethylamine then 9.8 mL methacryloyl chloride were added dropwise under vigorous stirring. The reaction mixture was allowed to warm to room temperature and stirred overnight. Then, the reaction was quenched with 30 mL of methanol and stirred for an hour. The solution was washed two times with distilled water and three times with brine. The separated organic phase was dried on anhydrous magnesium sulfate overnight. After filtration, the polymer solution was passed through a column filled with neutral aluminum oxide, and then, the solvent was removed by a rotary evaporator. The product was dried under vacuum until constant weight (yield: 88%). The obtained methacrylate-telechelic PTHF (MA-PTHF-MA) was characterized by ^1^H NMR. The molecular weight of the resulting MA-PTHF-MA with functionality of 2.0 is 2900 g/mol.

### 2.3. Synthesis of the PVIm-l-PTHF Polymer Conetworks

**Scheme 1:**
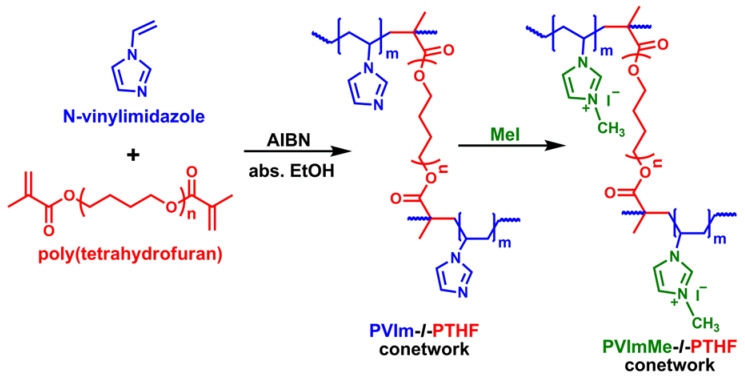
Synthesis of poly(1-vinylimidazole)-*l*-poly(tetrahydrofuran) conetworks by free radical copolymerization, and formation of poly(ionic liquid) conetworks (PIL-CNs) by alkylation with methyl iodide.

Poly(1-vinylimidazole)-*l*-poly(tetrahydrofuran) (PVIm-*l*-PTHF) conetworks were synthesized by free radical copolymerization of VIm and MA-PTHF-MA (Scheme 1). The free radical copolymerization of the VIm monomer and MA-PTHF-MA macro-crosslinker was carried out by using AIBN as the initiator in absolute ethanol as the solvent, as reported earlier [34]. The feed ratios of the MA-PTHF-MA and VIm reagents used for the conetwork syntheses were 50:50 and 60:40 wt/wt%. Briefly, the appropriate amounts of the MA-PTHF-MA crosslinker and VIm monomer were dissolved in ethanol in a glass vial, and then, the required amount of AIBN was added from a stock solution. The mixture was homogenized and deoxygenation was carried out with a nitrogen purge. The vials were thermostated at 65 °C for 3 days. The resulting conetworks were extracted first by THF, then by methanol, to remove the unreacted components and oligomers. The extraction was carried out in each solvent for 5 days, and the solvents were changed daily. Finally, the extracted conetworks were dried in vacuum at 120 °C until constant weight. Furthermore, the samples were annealed at 190 °C in a vacuum before every characterization measurements.

### 2.4. Alkylation of the Poly(1-vinylimidazole) with Methyl Iodide in the Conetworks

The synthesized conetworks were reacted with methyl iodide to convert the PVIm component to poly(ionic liquid)s. The conetwork samples were placed in a glass vial and a large excess of methyl iodide was added. After 3 days at room temperature, the swollen conetworks were removed from the vials, and the products were dried in a vacuum at 120 °C until constant weight.

### 2.5. Elemental Analysis

The composition of the conetworks was determined by elemental analysis. The measurements were made on a Heraeus CHN-O-RAPID instrument. The composition was calculated from the nitrogen content of the samples.

### 2.6. Solid-State NMR Measurements

Solid-state NMR magic angle spinning (MAS) spectra of the conetwork samples were recorded on a Varian NMR System 400 MHz with a Chemagnetics 4.0 mm narrow-bore double resonance T3 probe. The spinning rate of the rotor was 8 kHz in all cases. For the ^13^C CP MAS (cross polarization magic angle spinning) spectra, 12,000 transients were recorded with SPINAL-64 decoupling, 2 ms of contact time, and 5 s of recycle delay. The measuring temperature was 40 °C (ca. 15–35 °C above the melting temperature of PTHF), and the samples were incubated for 1 h before the measurements. Adamantane was used as the external chemical shift reference (38.55 and 29.50 ppm). The 90° pulse lengths were 2.9 μs for carbon and 2.9 μs for the proton channel.

### 2.7. Differential Scanning Calorimetry

The differential scanning calorimetry (DSC) measurements were carried out on a Mettler TG50 instrument under nitrogen atmosphere in the temperature range of −100 to 200 °C with 10 °C/min heating/cooling rate. On the recorded second heating curves, the glass transition temperature (*T*_g_) was determined as the inflection point of the DSC curve. The crystalline fraction (*X*_c_) was obtained from the integration of the endothermal peak.

### 2.8. Small-Angle X-ray Scattering

Small-angle X-ray scattering (SAXS) measurements were performed on the CREDO instrument equipped with a GeniX3D Cu ULD integrated beam delivery system having a 30W microfocus Cu-anode X-ray tube and a parabolic FOX3D graded multilayer mirror (Xenocs SA, Sassenage, France), as described elsewhere [84,85].

### 2.9. Atomic Force Microscopic Measurements

The atomic force microscopic (AFM) experiments were performed on a MultiMode scanning probe microscope with a Nanoscope IIIa controller (Digital Instruments) at ambient conditions in height and phase imaging modes. To guarantee a high resolution and avoid cantilever tip artefacts, new Nanosensors SSS cantilevers were used for these investigations. The flat cryo-sectioned surfaces of the annealed conetworks for these measurements were obtained by using a Diatome diamond knife at −100 °C with a Leica EMFCS microtome. It has to be noted that it is important to carry out the AFM measurements right after the cryo-microstomy, because the sectioned surfaces could adsorb some moisture within several hours and this may seriously affect the AFM imaging results.

### 2.10. Swelling Measurements

Weighted samples were placed in 50 mL of different solvents (distilled water, methanol, DMF, DMSO, hexane, THF, acetonitrile, N-methyl pyrrolidone, and ethyl acetate). Then, the weight of the samples was measured at predetermined time intervals for 3 days. The swelling degree (*Q*) was calculated by the following formula:

*Q* = (*m_t_* − *m*_0_) / *m*_0_ (1)

where *Q* is the swelling degree; *m*_0_ and *m_t_* are the weight of the initial dry and the swollen samples at a given swelling time (*t*), respectively.

## 3. Results and Discussion

### 3.1. Synthesis of and Alkylation in PVIm-l-PTHF Conetworks and Structural Characterizations

Two poly(1-vinylimidazole)-*l*-poly(tetrahydrofuran) (PVIm-*l*-PTHF) conetworks were synthesized by free radical copolymerization of 1-vinylimidazole (VIm) with methacrylate-telechelic poly(tetrahydrofuran) (MA-PTHF-MA) according to the literature procedure [34] as shown in Scheme 1. Copolymerizations with 50/50 and 60/40 wt/wt% feed ratios of MA-PTHF-MA/VIm resulted in conetworks with 85% and 77% gel fractions, respectively, indicating successful conetwork formation in both cases. Elemental analysis gave 57 and 76 wt% PTHF, and correspondingly 43 and 24 wt% PVIm content in these conetworks, respectively. These conetworks are denoted as PVIm-*l*-PTHF-57 and PVIm-*l*-PTHF-76, where the numbers stand for the PTHF contents (wt%) in these materials. It has to be noted that the PTHF crosslinker content is higher in the conetworks than in the feed, which is in line with previous reports [34,35], according to which both the reactivity ratio differences between methacrylates and 1-vinylimidazole, and the uneven partitioning of VIm between the region of the chain ends surrounded by the hydrophobic PTHF and the solution, lead to this effect. As shown later in this study (see Figures 3‒5), in accordance with a previous report [39], nanophase-separated PVIm-*l*-PTHF conetworks have been obtained.

**Figure 1:**
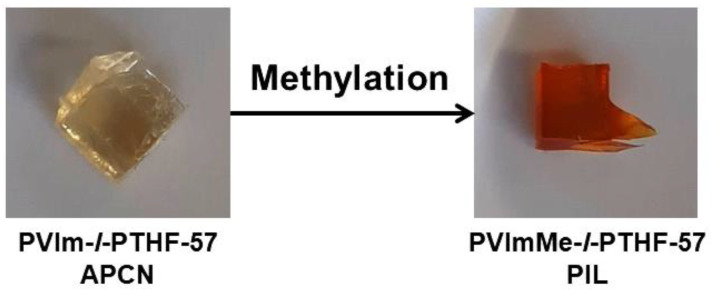
Pictures of the PVIm-*l*-PTHF conetwork before and after methylation with methyl iodide.

Converting the PVIm chains in the PVIm-*l*-PTHF conetworks to imidazolium containing PIL was attempted by alkylation of the imidazole rings in the PVIm chains with methyl iodide (MeI), as displayed in Scheme 1. For this reaction, in the PVIm nanophases, the conetworks were swollen with excess of MeI. After three days of this treatment, the samples were removed from the MeI solvent, and the weights of the resulting swollen materials were measured. The gained weights were found 601% and 275% for the PVIm-*l*-PTHF-57 and PVIm-*l*-PTHF-76 conetworks, respectively, indicating high swelling ability of the conetworks in MeI. Subsequently, the samples were dried under vacuum until constant weight to remove the unreacted MeI in order to determine the extent of alkylation. On the basis of the weight increase by the treatment with MeI, it was found that the extents of alkylation in the PVIm-*l*-PTHF-57 and PVIm-*l*-PTHF-76 conetworks were 93% and 96%, respectively. These findings indicate that independent of the PVIm content of the conetworks, nearly quantitative methylation was achieved in the nanophase-separated PVIm-*l*-PTHF conetworks. As shown in Figure 1, the color of the alkylated sample changed to orange from the pale yellow of the starting conetwork by reacting it with MeI, indicating structural changes, i.e., alkylation of the imidazole moieties in the conetworks. The methylated conetworks are denoted furthermore as PVImMe-*l*-PTHF-57 and PVImMe-*l*-PTHF-76.

**Figure 2:**
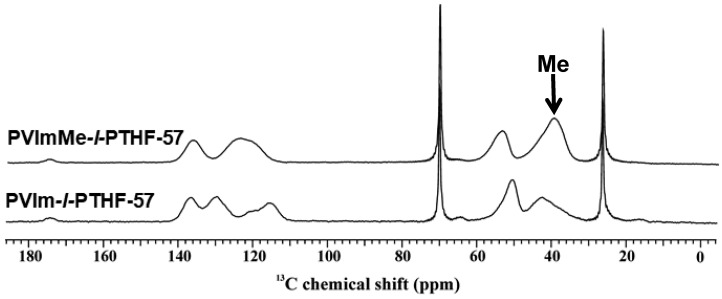
Solid-state ^13^C NMR spectra of the PVIm*-l*-PTHF-57 and the methylated PVImMe*-l*-PTHF-57 poly(ionic liquid) conetworks.

The successful methylation of the PVIm chains in the PVIm-*l*-PTHF conetworks is also supported by solid-state ^13^C CP MAS analyses. As shown in Figure 2, the signals in the aromatic region (110–140 ppm in the ^13^C NMR spectra) are changed clearly, indicating the effect of the methylation of the nitrogen in the imidazole rings. Because the signals of the unmethylated rings cannot be observed in this spectrum, taking into account the accuracy of the measurements, we can conclude that the methylation efficiency was at least 95%, which corresponds well with the methylation yields determined gravimetrically. The *N*-methyl signals are also identifiable at 39 ppm in the NMR spectrum of the PVImMe-*l*-PTHF conetwork. It is also noteworthy that the signals belonging to the main chain carbon atoms of PVIm (32–60 ppm) also show some changes in the ^13^C NMR spectra. These changes may originate from the modifications of the main chain conformation caused by ionic forces. Because the signals of the PTHF crosslinker do not show differences at 26.1 ppm and at 70.0 ppm, it is evident that the PTHF chains are unaffected by the methyl iodide treatment of the PVIm-*l*-PTHF conetworks. On the basis of these and the gravimetric data, it can be claimed that the applied alkylation method leads with nearly quantitative yields to poly(3-methyl-1-vinylimidazolium iodide)-*l*-poly(tetrahydrofuran) poly(ionic liquid) conetworks (PIL-CNs).

**Figure 3:**
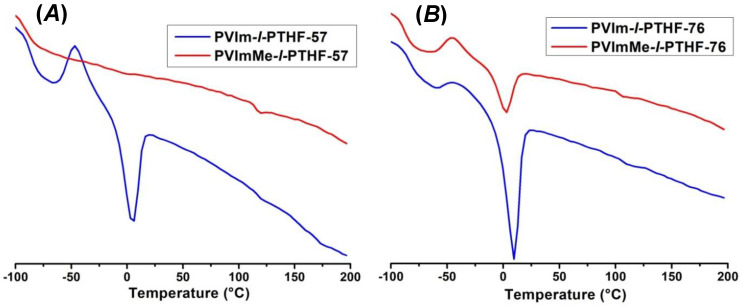
DSC curves of the PVIm-*l*-PTHF-57 (**A**) and PVIm-*l-*PTHF-76 (**B**) (blue lines) and the methylated PVImMe-*l*-PTHF-57 (**A**) and PVImMe*-l*-PTHF-76 (**B**) poly(ionic liquid) (red lines) conetworks.

The DSC curves of the PVIm-*l*-PTHF conetworks and the methylated PVImMe-*l*-PTHF derivatives are displayed in Figure 3, and the obtained glass transition temperature (*T*_g_), melting temperature (*T*_m_), and crystalline fraction (*X*_c_) data are shown in Table 1. For evaluating these data, it has to be kept in mind that the PVIm homopolymer has a *T*_g_ at 171 °C, while the *T*_g_ of the MA-PTHF-MA crosslinker is −81 °C, and its melting temperature is 25 °C with 42% crystalline fraction. As can be clearly seen in Figure 3 and in Table 1, all the conetworks, including the PVImMe-*l*-PTHF-57 and PVImMe-*l*-PTHF-76 PIL-CNs as well, have two *T*_g_s, one in the region of the *T*_g_ of the PTHF homopolymer, and another one in the region of 103–116 °C for the PVIm and PVImMe components. The observed two *T*_g_s in these conetworks indicate clear evidence that the chemically crosslinked immiscible PTHF and PVIm, and PTHF and PVImMe components as well, form two distinct phases in both the PVIm-*l*-PTHF and PVImMe-*l*-PTHF conetworks, respectively. It has to be emphasized that these findings mean that the phase-separated structure is maintained even after the alkylation reaction as well in the methylated conetworks. As shown in Figure 3 and Table 1, the *T*_g_ of the PTHF phase does not change considerably in the conetworks upon methylation, which corroborates the findings of the solid-state NMR analysis. The *T*_g_s of the PVIm component are significantly lower in the conetworks than that of the PVIm homopolymer. This is in accordance with the so called “scissor effect” in conetworks [37], according to which the decrease in the *T*_g_ of the crosslinked chains in conetworks is related to the molecular weight of the PVIm chains between two crosslinking points in the conetworks. The formation of the ionic liquid moiety leads only to a small degree of *T*_g_ decrease, indicating that the alkylation of the PVIm chains does not have a remarkable effect on the chain segment mobility of the main chain. In contrast, significant changes occur in relation to the melting temperature and crystalline fraction of the PTHF phase in these conetworks. As already reported [37], both the *T*_m_ and *X*_c_ are smaller in the PVIm-*l*-PTHF conetworks than that of the PTHF homopolymer. Remarkably, the crystalline fraction of PTHF completely disappears by methylation of the PVIm-*l*-PTHF-57 conetwork, while both the *T*_m_ and *X*_c_ are smaller in the PVImMe-*l*-PTHF-76 poly(ionic liquid) conetwork than in the unmethylated PVIm-*l*-PTHF-76 precursor. These changes can also be considered as indications of the successful formation of the poly(ionic liquid) phase in the conetworks by the applied alkylation process.

**Figure 4:**
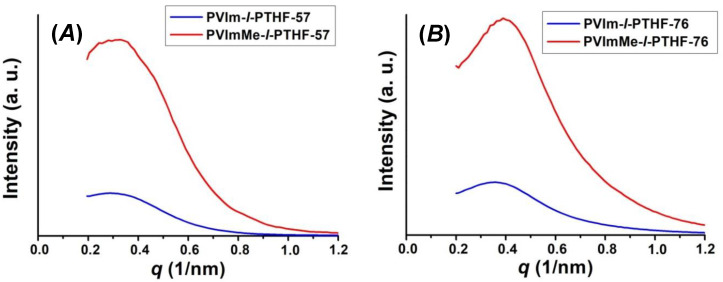
SAXS curves of the PVIm-*l*-PTHF-57 (**A**) and PVIm-*l-*PTHF-76 (**B**) (blue lines) and the methylated PVImMe-*l*-PTHF-57 (**A**) and PVImMe*-l*-PTHF-76 (**B**) poly(ionic liquid) (red lines) conetworks.

In order to reveal the effect of alkylation of the PVIm chains on the phase-separated structure, small angle X-ray scattering (SAXS) and atomic force microscopy (AFM) measurements were carried out with the PVIm-*l*-PTHF conetworks and the PVImMe-*l*-PTHF PIL-CN derivatives. As shown in Figure 4, a scattering curve with one maximum is observed, indicating disordered phase separation in all cases, including the PVImMe-*l*-PTHF PIL-CNs as well. This corroborates the result of previous morphology investigations on the PVIm-*l*-PTHF conetworks [39]. In addition, the scattering intensities of the PVImMe-*l*-PTHF conetworks are significantly higher than that of the corresponding PVIm-*l*-PTHF samples. This is due to the presence of the iodide ion in the methylated PIL phase. It is also important to note that the peak maximum as a function of the scattering vector (*q*) does not change substantially by methylation of the conetworks. Evidently, this means that the PVIm-*l*-PTHF conetworks keep their nanophase-separated morphology in the alkylated PIL conetworks as well. This is well reflected by comparing the d-spacing values (*d* = 2π/*q*_max_). As shown in Table 1, the d-spacings are 21.9 and 20.1 nm for the PVIm-*l*-PTHF-57 and the PVImMe-*l*-PTHF-57 conetworks, respectively. The PVIm-*l*-PTHF-76 and PVImMe-*l*-PTHF-76 samples have 17.6 and 15.7 nm d-spacing values, respectively. As found earlier [39], the domain spacing decreases with increasing crosslinker content in the PVIm-*l*-PTHF conetworks, and the d-spacing values indicate the same relationship for the PIL-CN conetworks as well, on the one hand. On the other hand, it is evident from the d-spacing values that methylation of the PVIm chains results in somewhat smaller average domain distances than that observed in the unmethylated conetworks, i.e., the d-spacings are 21.9, 20.1, 17.6, and 15.7 nm in the PVIm-*l*-PTHF-57, PVImMe-*l*-PTHF-57, PVIm-*l*-PTHF-76, and PVImMe-*l*-PTHF-76 conetworks, respectively. The lack of expected d-spacing increase as a consequence of PIL formation in the conetworks can be attributed to the relatively high crosslinking densities in these conetworks, and also to the strong hydrogen bonding of the iodide ion with the hydrogens of the alkyl imidazolium rings [86,87]. Consequently, the results of the SAXS measurements indicate that the reaction of MeI with the PVIm chains in the nanophase-separated PVIm-*l*-PTHF conetworks leads to poly(ionic liquid) conetworks with d-spacing of ~15–20 nm having methylated poly(3-methyl-1-vinylimidazolium iodide) PIL and PTHF nanophases.

**Figure 5:**
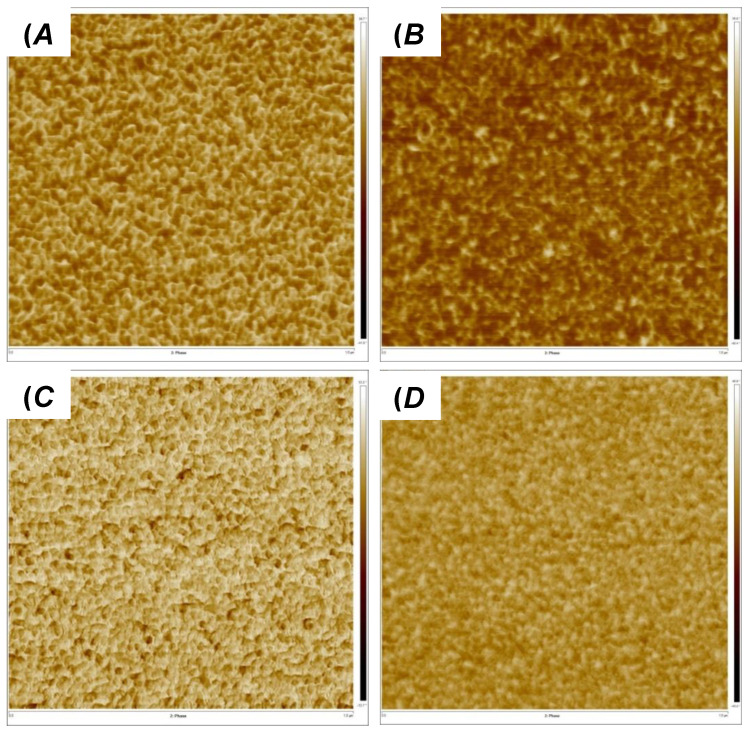
AFM images of the PVIm-*l*-PTHF-57 (**A**), PVIm-*l-*PTHF-76 (**B**), PVImMe-*l*-PTHF-57 (**C**), and PVImMe*-l*-PTHF-76 (**D**) conetworks (1000 × 1000 nm^2^).

As displayed in Figure 5, the phase mode AFM images further corroborate the nanophase-separated morphologies of both the PVIm-*l*-PTHF and the PVImMe-*l*-PTHF PIL conetworks. In these images, the darker regions correspond to the softer PTHF, while the white areas belong to the harder PVIm and PVImMe phases. The nanophase-separated morphologies are clearly visible in these images both before (Figure 5A,B) and after (Figure 5C,D) the alkylation process. In other words, the poly(ionic liquid) formation from the PVIm nanophases keeps the nanophase-separated structure of the conetworks, i.e., PIL conetworks with PVImMe and PTHF nanophases are formed. Evaluation of the AFM images allows for the estimation of the average phase sizes and the average domain distances in the conetworks. As presented in Table 1, the average domain sizes of the PTHF phases (*d*_PTHF_) decrease from 15.0 to 13.5 nm and from 18.2 to 9.0 nm as a result of the methylation in the PVIm-*l*-PTHF-57 and PVIm-*l*-PTHF-76 conetworks, respectively. In contrast, a slight increase is obtained in the domain sizes from 7.8 nm for the PVIm to 8.4 nm for the PVImMe, and from 7.9 nm for the PVIm to 8.2 nm for the PVImMe phases upon alkylation with methyl iodide in the PVIm-*l*-PTHF-57 and PVIm-*l*-PTHF-76 conetworks, respectively. These findings can be attributed to the volume increase by methylation of the PVIm phase. As already mentioned in relation to the SAXS results above, the lack of larger domain size increase can be attributed to the crosslinked structure of these conetworks and also to the hydrogen bonding between the imidazolium rings and iodide ions, which is in close proximity of the iodide ions to the imidazolium rings, as reported for low molecular weight ionic liquids with halogen counter ions [86,87]. The decrease in the domain sizes of the elastic PTHF phases in the PIL-CNs can be explained by the compression (hardening) effect of the harder PIL phases in comparison to the PVIm phases in the PVIm-*l*-PTHF conetworks. It must be mentioned that the image contrast in phase imaging responds not only to the nanosized soft/hard areas but also to the overall hardness averaged throughout a surface of a few hundred nanometers. Nonetheless, the average domain distances could be estimated from the AFM images and these fit relatively well with that of the SAXS measurements as shown in Table 1. Based on the AFM and SAXS results, it can be concluded that the nanoconfined phase-separated morphology of the PVIm-*l*-PTHF conetworks is kept even after the highly efficient alkylation of the PVIm nanophases, that is, poly(ionic liquid) conetworks with nanocompartmentalized poly(3-methyl-1-vinylimidazolium iodide) PIL domains with ~8–9 nm average domain sizes are formed by the applied alkylation process.

### 3.2. Swelling Properties of the PVIm-l-PTHF Amphiphilic and PVImMe-l-PTHF Poly(ionic liquid) Conetworks

**Figure 6:**
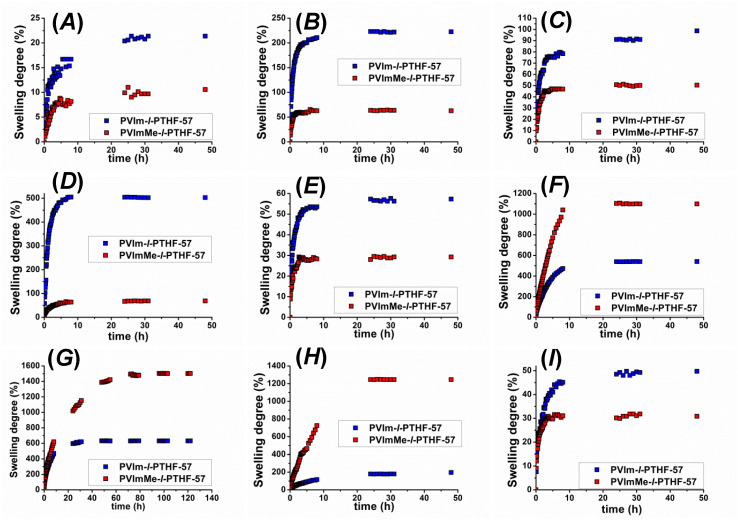
The swelling degrees as a function of time of the PVIm-*l*-PTHF-57 (blue) and PVImMe-*l*-PTHF-57 poly(ionic liquid) (red) conetworks as a function of time in different solvents: hexane (**A**), THF (**B**), ethyl acetate (**C**), methanol (**D**), acetonitrile (**E**), DMF (**F**), NMP (**G**), DMSO (**H**), and distilled water (**I**).

**Figure 7:**
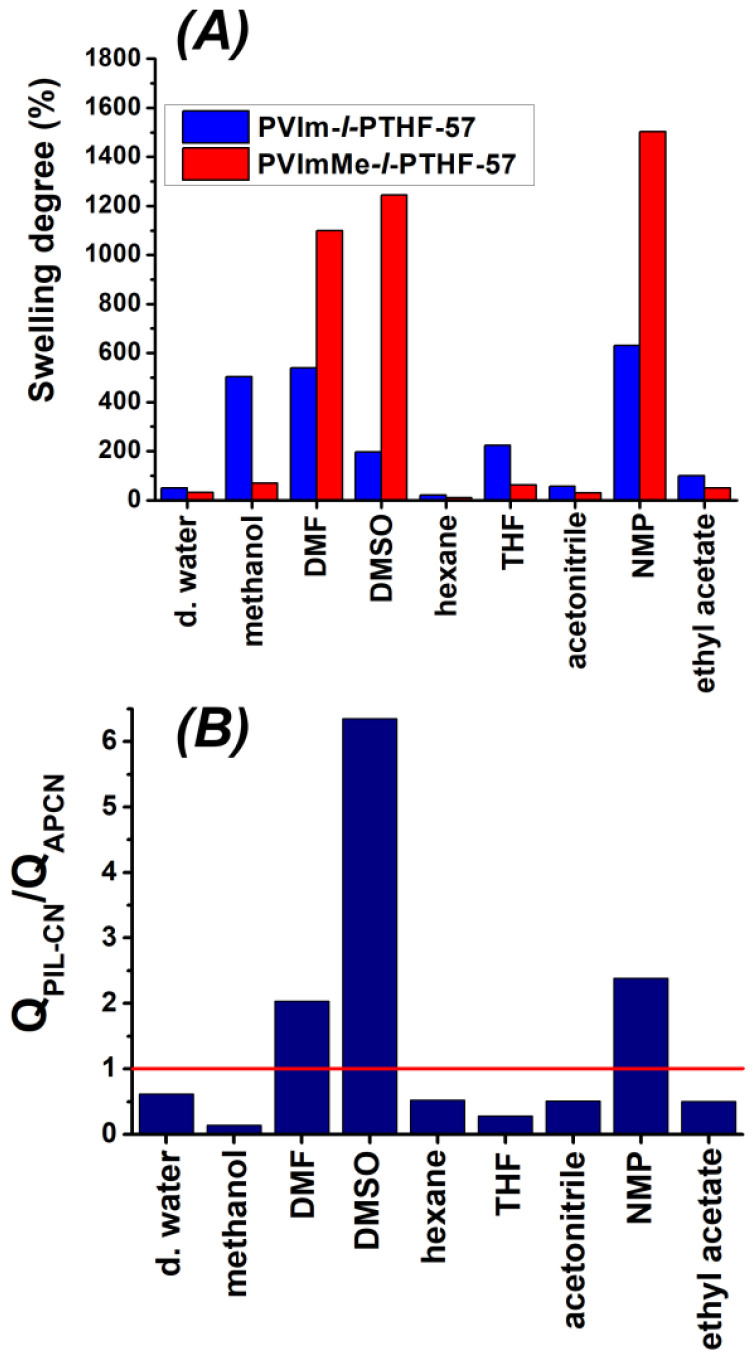
Equilibrium swelling degrees (**A**) and the ratio of the equilibrium swelling degrees (**B**) of PVIm*-l-*PTHF-57 (*Q*_APCN_) and PVImMe*-l-*PTHF-57 poly(ionic liquid) (*Q*_PIL-CN_) conetworks in different solvents (the red line indicates ratio of one).

Considering that the swelling properties of crosslinked polymers are of paramount importance, detailed swelling investigations with the PVIm-*l*-PTHF APCNs and PVImMe-*l*-PTHF PIL-CNs were carried out by us with a broader range of widely used polar and nonpolar solvents, including ethyl acetate (EtOAc), N-methylpyrrolidone (NMP), acetonitrile (ACN), THF, hexane (Hex), DMSO, DMF, methanol (MeOH), and distilled water. As reported earlier, the PVIm-*l*-PTHF conetworks have amphiphilic swelling characteristics [34], i.e., these conetworks swell in both hydrophilic and hydrophobic solvents. Indeed, this is well demonstrated in Figure 6, which shows the swelling degrees as a function of time for both kinds of conetworks. The PVIm-*l*-PTHF conetwork swells in the hydrophobic THF and hexane, and in the hydrophilic water and methanol as well, for instance, the equilibrium swelling degrees are 503% and 223% in methanol and THF, respectively. The quaternized PVImMe-*l*-PTHF conetwork also possesses amphiphilic character. However, even the first look at the data in Figure 6 indicates that there are significant differences between the swelling capacities of the PVIm-*l*-PTHF APCN and the PVImMe-*l*-PTHF PIL-CN. As displayed in Figure 7A and shown in Table 2, the equilibrium swelling degrees (*Q*) of the PIL-CN, which is a polyelectrolyte, are surprisingly lower in water and methanol, and in acetonitrile and ethyl acetate as well, than that of the PVIm-*l*-PTHF APCN, on the one hand. On the other hand, the equilibrium swelling degrees of the PVImMe-*l*-PTHF PIL-CN is dramatically higher in DMF, DMSO, and NMP, than that of the starting APCN. It is also remarkable that the PVImMe-*l*-PTHF-57 PIL-CN is able to absorb more than 1000% liquids in DMF, DMSO, and NMP, and as such, this PIL-CN can be considered as a superabsorbent for these solvents.

The ratios of the equilibrium swelling degrees for the PVImMe-*l*-PTHF PIL-CN (*Q*_PIL-CN_) and the PVIm-*l*-PTHF APCN (*Q*_APCN_) are depicted in Figure 7B. The quaternization decreases the *Q* not only in the aprotic hydrophobic solvents, i.e., by about 70% in THF and 50% in hexane, but also in the polar acetonitrile and ethyl acetate as well. Surprisingly, this trend can also be observed in polar protic solvents, such as MeOH and water. The equilibrium swelling degree is reduced by about 40% in water and to nearly one-tenth in methanol. In large contrast, the swelling capacity of the PIL-CN increases by more than two times in DMF and NMP, and a more than sixfold increase is observed in DMSO. This unprecedented selective swelling behavior of the nanoconfined PVImMe-*l*-PTHF PIL-CN can be considered to be utilized for several application possibilities, such as catalysis purposes, solvent purification, and environmental protection processes, etc.

**Figure 8:**
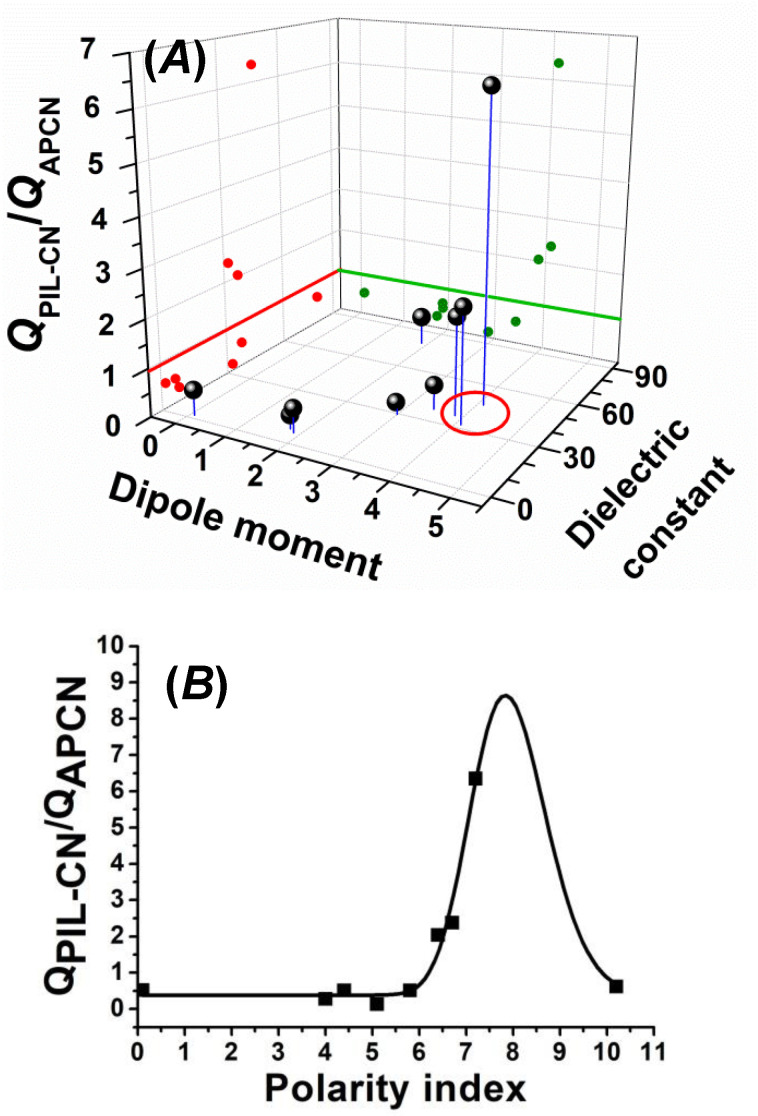
The ratio of the equilibrium swelling degrees of the PVImMe*-l-*PTHF-57 poly(ionic liquid) conetwork (Q_PIL-CN_) and the PVIm*-l-*PTHF-57 (Q_APCN_) conetwork as a function of the dipole moment and dielectric constant of the solvents (**A**), and as a function of the polarity index of the solvents (**B**).

Finding a relationship between the observed changes in the swelling behavior upon the formation of the PIL phase in the PVIm-*l*-PTHF conetwork and fundamental solvent polarity characteristics, such as dielectric constant, dipole moment, and Snyder’s polarity index [88,89], was also attempted. In this context, it has to be mentioned that only one study [69] was found by us which reported on the correlation between the equilibrium swelling degree and dielectric constant for crosslinked poly(3-alkyl-1-vinylimidazolium-*co*-acrylic acid) PILs, in which both the imidazolium cations and carboxylate counter ions are located in the same copolymer chain. In contrast, we have found that no characteristic relationship exists between the equilibrium swelling degrees of the PVIm-*l*-PTHF APCN or the PVImMe-*l*-PTHF PIL-CN and the dielectric constant, dipole moment, or polarity index of the investigated solvents. The data in Table 2 indicate only that solvents with higher than a 3.7–3.8 D dipole moment may result in high swelling capacities of the PVImMe-*l*-PTHF PIL-CN. However, displaying the *Q*_PIL-CN_/*Q*_APCN_ ratios in a three-dimensional plot as a function of the dipole moment and dielectric constant (Figure 8A) shows that a tight dipole moment–dielectric constant region exists where the three solvents, i.e., DMSO, DMF, and NMP with a high degree of relative swelling, are located. Namely, when the dipole moment of the applied solvent is higher than 3.8 and the dielectric constant is between 30 and 50, the ratios of *Q*_PIL-CN_/*Q*_APCN_ increase up to 2 to 6 times, but otherwise, these swelling ratios are lower than one. Unexpectedly, plotting the *Q*_PIL-CN_/*Q*_APCN_ data as a function of the polarity index, a Gaussian curve can be fitted for the equilibrium swelling ratios. This finding indicates that solvents falling in a relatively narrow polarity index region, roughly in ~6–9.5, are able to swell the PIL-CNs with high *Q*_PIL-CN_/*Q*_APCN_ equilibrium swelling ratios, and the swelling capacity of solvents with polarity index below and above this region becomes low, like in the case of methanol and water with a polarity index of 5.1 and 10.2, respectively.

Evaluation of the swelling degrees versus time plots was carried out by the Korsmeyer–Peppas relationship [90] as well, which provides information about the solvent diffusion process in the conetworks:

*R = k t^n^*

where *R* stands for the ratio of the swelling degree at times *t* and the equilibrium swelling degree, and *k* and *n* are constants. The type of the diffusion process of the solvents can be classified by the value of the *n* exponent obtained by the double logarithmic plot on the basis of the above equation, i.e., log(*R*) versus log(*t*). When *n* is close to 0.5 or smaller, then the swelling occurs mainly by Fickian diffusion, while higher values indicate that other processes, e.g., relaxation phenomena and/or strong solvent–network interactions, influence also the solute diffusion in the conetworks, that is, non-Fickian diffusion, and so called anomalous transport takes place. As shown in Table 3, with the exception of DMF, DMSO, and NMP, the *n* values are in the range of 0.5 or somewhat smaller, implying swelling by Fickian diffusion by the other solvents, i.e., water, methanol, hexane, THF, acetonitrile, and ethyl acetate. With the PVImMe-*l*-PTHF PIL-CN, 0.64, 0.71, and 0.63 *n* values are obtained for DMF, DMSO, and NMP, respectively. These findings evidently indicate that anomalous swelling, i.e., non-Fickian diffusion, occurs with these solvents for the PVImMe-*l*-PTHF poly(ionic liquid) conetworks. These results also allow for the conclusion to be made that presumably preferential interaction exists between these solvents and the nanoconfined PVImMe-*l*-PTHF PILs. The data in Table 3 also show that the *n* values are somewhat higher than 0.5, even in the case of PVIm-*l*-PTHF for swelling in DMF, DMSO, and NMP. These are in line with the observed high swelling capacity of both the PVIm-*l*-PTHF, and especially the PVImMe-*l*-PTHF nanoconfined poly(ionic liquid) conetwork for these solvents.

## 4. Conclusions

A new type of nanoconfined poly(ionic liquid) conetworks (PIL-CNs), which has not existed before according to the best of our knowledge, was successfully synthesized by a simple alkylation process of the pendant imidazole rings of poly(1-vinylimidazole) with methyl iodide in bicontinuous nanophasic poly(1-vinylimidazole)-*l*-poly(tetrahydrofuran) (PVIm-*l*-PTHF) amphiphilic conetworks (APCNs). Gravimetric and solid-state NMR measurements indicate that the yields of the alkylation reaction in the conetworks were over 95%, and poly(3-methyl-1-vinylimidazolium iodide) (PVImMe) poly(ionic liquid) phases, crosslinked with PTHF chains, are formed. DSC analysis showed two distinct glass transitions in both the APCNs and PIL-CNs, indicating phase-separated morphology even after the alkylation process as well, i.e., the PVImMe PIL phase and the PTHF crosslinker are immiscible even at the nanoscale. Significant suppression of crystallinity of the PTHF component in the conetworks was found as a result of alkylation. In conetworks with higher PVIm content, the crystallinity fully disappears after the alkylation reaction, i.e., the charged PVImMe PIL chains prevent crystal growth nucleation of the semicrystalline PTHF. SAXS and AFM measurements clearly indicate bicontinuous nanophase-separated morphology in both the PVIm-*l*-PTHF and PVImMe-*l*-PTHF poly(ionic liquid) conetworks with domain sizes nearly in the same range. This means that the formation of PVImMe PIL phases by alkylation of the PVIm nanophases does not destroy the mutually nanoconfined morphology of the PVImMe PIL and PTHF phases in these conetworks. Evaluation of the SAXS curves with distinct peak maxima and the phase mode AFM images indicates disordered bicontinuous (cocontinuous) nanophase-separated structure in both the PVIm-*l*-PTHF and PVImMe-*l*-PTHF poly(ionic liquid) conetworks with 22–26 and 17–22 nm domain spacing, respectively, on the one hand. On the other hand, the domain sizes of the PVIm and PVImMe phases in the PVIm-*l*-PTHF and PVImMe-*l*-PTHF poly(ionic liquid) conetworks obtained by AFM fall in the ranges of 7.8–7.9 and 8.2–8.4 nm. The lack of the expected higher average domain size increase in the PVImMe-based phases as a result of alkylation can be attributed to the relatively high crosslinking densities and the known strong hydrogen bonding between the iodide ions and the imidazolium rings in these PIL-CNs. In contrast, shrinkage of the PTHF phase occurs by methylation from 15–19 nm in the PVIm-*l*-PTHF to 9–14 nm in the PVImMe-*l*-PTHF poly(ionic liquid) conetworks. Presumably, this is due to the compression effect of the ionic PIL phases on the elastic hydrophobic PTHF nanophases. Based on these findings, it can be concluded that the applied robust alkylation process is suitable to convert the PVIm nanophases in PVIm-*l*-PTHF conetworks to PVImMe poly(ionic liquid) nanophases without the loss of the bicontinuous nanoconfined morphology of these conetworks. In other words, PVImMe-*l*-PTHF poly(ionic liquid) conetworks with distinct nanocompartmentalized PVImMe phases are formed with average domain sizes in the range of 8.2–8.4 nm.

Detailed comprehensive swelling studies with the PVIm-*l*-PTHF and PVImMe-*l*-PTHF poly(ionic liquid) conetworks using nine different swelling agents, such as water, methanol, THF, DMSO, DMF, NMP, hexane, ethyl acetate, and acetonitrile, covering a wide range of philicity and polarity characteristics, resulted in surprising unprecedented results. Although, the PIL component in the PVImMe-*l*-PTHF PIL-CNs is a polyelectrolyte, it was found that the equilibrium swelling degrees are smaller in the PVImMe-*l*-PTHF than in the PVImMe-*l*-PTHF PIL conetworks, not only for the less polar hydrophobic solvents, i.e., hexane, THF and ethyl acetate, but for acetonitrile, and even for the hydrophilic protic water and methanol as well. In contrast, unexpectedly higher than 1000% equilibrium swelling degrees were observed for the PVImMe-*l*-PTHF PIL-CN in the aprotic polar DMSO, DMF, and NMP, providing superabsorbent behavior selectively for these solvents. The swelling capacity of the PVImMe-*l*-PTHF PIL-CN is more than one order of magnitude higher for these three solvents than that for the rest of the investigated swelling agents, for which the swelling degrees fall only in the 11–69% range. Searching for a correlation between solvent characteristics and the observed unusual swelling of the PVImMe-*l*-PTHF PIL-CN in the examined solvents, one can conclude that there is no direct correlation between the absolute equilibrium swelling ratios of either the PVIm-*l*-PTHF or the PVImMe-*l*-PTHF PIL conetworks and dielectric constants or dipole moments of the solvents. The data only indicate that high, selective superabsorbent behavior of the PIL-CN can be expected for solvents with higher than about 3.6–3.7 D dipole moments. Interestingly, however, the ratio of the equilibrium swelling degrees (*Q*_PIL-CN_/*Q*_APCN_) of the two types of conetworks as a function of the polarity index of the solvents can be fitted with a Gaussian curve with a maximum at 7.8. This dependence of *Q*_PIL-CN_/*Q*_APCN_ on the polarity index also shows that increased swelling of the PVImMe-*l*-PTHF PIL-CNs can be expected only with solvents having polarity index values in the ~6–9.5 range.

In sum, the robust process applied by us, i.e., soaking PVIm-*l*-PTHF conetworks in MeI, leads to a high extent of alkylation to form novel PVImMe-*l*-PTHF PIL-CNs with nanoconfined PIL phases. These new materials possess unprecedented unique swelling properties with selective superabsorbent properties for polar aprotic solvents with a range of polarity index of 6–9.5, such as DMF, DMSO, and NVP. These fundamental swelling properties and the potential of ion exchange of the counter ion in these PIL-CNs is expected to lead to new research and development directions with such PIL containing materials, and enable the application of the resulting PIL-CNs in broad application fields, for example, nanohybrid syntheses, biomedicine, catalysis, solvent and wastewater purification, gas sorption membranes (e.g., CO_2_), and ion or proton conduction for batteries and fuel cells, respectively, which will be investigated in our future research.

## Figures and Tables

**Table 1:** The composition of the PVIm-*l-*PTHF and PVImMe-*l-*PTHF conetworks, yield of alkylation, the glass transition temperature (*T*_g_) of the PTHF, PVIm, and PVImMe^+^I^−^ components, the melting temperature (*T*_m_) and crystalline fraction (*X*_c_), the *d*-spacing, the average domain sizes (*d*_PTHF_, *d*_PVIm_, and *d*_PVImMe_), and the average domain distances in the corresponding PVIm*-l-*PTHF and PVImMe*-l-*PTHF conetworks.

	PVIm-*l*-PTHF-57	PVImMe-*l*-PTHF-57	PVIm*-l*-PTHF-76	PVImMe-*l*-PTHF-76
PTHF content (wt%)	57	36	76	56
PVIm content (wt%)	43	2	24	1
PVImMe^+^I^−^ content (wt%)	-	62	-	43
*T*_g_ of PTHF (°C) ^(a)^	−86	−89	−83	−87
*T*_g_ of PVIm and PVImMe (°C) ^(b)^	116	114	106	103
*T*_m_ of PTHF (°C) ^(c)^	4.8	-	10.0	2.7
*X*_c_ of PTHF (%) ^(d)^	9.4	0.0	13.7	5.6
*d*-spacing by SAXS (nm)	21.9	20.1	17.6	15.7
*d*_PTHF_ (nm) ^(e)^	15.0	13.5	18.5	9.0
*d*_PVIm_ and *d*_PVImMe_ (nm) ^(e)^	7.8	8.4	7.9	8.2
average domain distance (nm) ^(e)^	22.8	21.9	26.1	17.2

(**a**) *T*_g_(PTHF) = −81 °C, (**b**) *T*_g_(PVIm) = 176 °C, (**c**) *T*_m_(PTHF) = 25 °C, (**d**) *X*_c_(PTHF) = 42%, (**e**) by phase mode AFM.

**Table 2:** The equilibrium swelling degrees of the PVIm*-l-*PTHF-57 (*Q*_APCN_) and PVImMe*-l-*PTHF-57 (*Q*_PIL-CN_) conetworks, the ratio of their swelling degrees (*Q*_PIL-CN_/*Q*_APCN_), and the dipole moment, dielectric constant, and polarity index of the investigated solvents.

Solvent	*Q*_APCN_ (%)	*Q*_PIL-CN_ (%)	*Q*_PIL-CN_/*Q*_APCN_	Dipole Moment (D)	Dielectric Constant	Polarity Index
water	50	31	0.62	1.87	80.10	10.2
methanol	503	69	0.14	2.87	32.70	5.1
DMF	540	1099	2.03	3.86	36.71	6.4
DMSO	196	1245	6.35	4.10	46.68	7.2
hexane	21	11	0.52	0.08	1.88	0.1
THF	223	63	0.28	1.75	7.58	4.0
acetonitrile	57	29	0.51	3.44	37.50	5.8
NMP	630	1503	2.38	4.09	32.20	6.7
ethyl acetate	99	50	0.50	1.88	6.02	4.4

**Table 3:** The exponents of the swelling degree versus time curves obtained by the Korsmeyer–Peppas relationship for the PVIm-*l*-PTHF-57 (*n*_APCN_) and PVImMe-*l*-PTHF-57 (*n*_PIL-CN_) conetworks swollen in different solvents.

Solvent	*n*_APCN_	*n*_PIL-CN_
water	0.43	0.36
methanol	0.36	0.46
DMF	0.58	0.64
DMSO	0.56	0.71
hexane	0.38	0.49
THF	0.52	0.52
acetonitrile	0.48	0.34
N-methylpyrrolidone	0.58	0.63
ethyl acetate	0.46	0.50
